# GPro: generative AI-empowered toolkit for promoter design

**DOI:** 10.1093/bioinformatics/btae123

**Published:** 2024-03-01

**Authors:** Haochen Wang, Qixiu Du, Ye Wang, Hanwen Xu, Zheng Wei, Xiaowo Wang

**Affiliations:** Ministry of Education Key Laboratory of Bioinformatics, Tsinghua University, Beijing 100084, China; Center for Synthetic and Systems Biology, Tsinghua University, Beijing 100084, China; Beijing National Research Center for Information Science and Technology, Tsinghua University, Beijing 100084, China; Department of Automation, Tsinghua University, Beijing 100084, China; Ministry of Education Key Laboratory of Bioinformatics, Tsinghua University, Beijing 100084, China; Center for Synthetic and Systems Biology, Tsinghua University, Beijing 100084, China; Beijing National Research Center for Information Science and Technology, Tsinghua University, Beijing 100084, China; Department of Automation, Tsinghua University, Beijing 100084, China; Ministry of Education Key Laboratory of Bioinformatics, Tsinghua University, Beijing 100084, China; Center for Synthetic and Systems Biology, Tsinghua University, Beijing 100084, China; Beijing National Research Center for Information Science and Technology, Tsinghua University, Beijing 100084, China; Department of Automation, Tsinghua University, Beijing 100084, China; Ministry of Education Key Laboratory of Bioinformatics, Tsinghua University, Beijing 100084, China; Center for Synthetic and Systems Biology, Tsinghua University, Beijing 100084, China; Beijing National Research Center for Information Science and Technology, Tsinghua University, Beijing 100084, China; Department of Automation, Tsinghua University, Beijing 100084, China; Ministry of Education Key Laboratory of Bioinformatics, Tsinghua University, Beijing 100084, China; Center for Synthetic and Systems Biology, Tsinghua University, Beijing 100084, China; Beijing National Research Center for Information Science and Technology, Tsinghua University, Beijing 100084, China; Department of Automation, Tsinghua University, Beijing 100084, China; Ministry of Education Key Laboratory of Bioinformatics, Tsinghua University, Beijing 100084, China; Center for Synthetic and Systems Biology, Tsinghua University, Beijing 100084, China; Beijing National Research Center for Information Science and Technology, Tsinghua University, Beijing 100084, China; Department of Automation, Tsinghua University, Beijing 100084, China

## Abstract

**Motivation:**

Promoters with desirable properties are crucial in biotechnological applications. Generative AI (GenAI) has demonstrated potential in creating novel synthetic promoters with significantly enhanced functionality. However, these methods' reliance on various programming frameworks and specific task-oriented contexts limits their flexibilities. Overcoming these limitations is essential for researchers to fully leverage the power of GenAI to design promoters for their tasks.

**Results:**

Here, we introduce GPro (Generative AI-empowered toolkit for promoter design), a user-friendly toolkit that integrates a collection of cutting-edge GenAI-empowered approaches for promoter design. This toolkit provides a standardized pipeline covering essential promoter design processes, including training, optimization, and evaluation. Several detailed demos are provided to reproduce state-of-the-art promoter design pipelines. GPro's user-friendly interface makes it accessible to a wide range of users including non-AI experts. It also offers a variety of optional algorithms for each design process, and gives users the flexibility to compare methods and create customized pipelines.

**Availability and implementation:**

GPro is released as an open-source software under the MIT license. The source code for GPro is available on GitHub for Linux, macOS, and Windows: https://github.com/WangLabTHU/GPro, and is available for download via Zenodo repository at https://zenodo.org/doi/10.5281/zenodo.10681733.

## 1 Introduction

Synthetic promoters play important roles in a variety of bioengineering applications, providing precise control over gene transcriptional regulation (Khalil and Collins 2010). Recently, by employing deep learning-based generative models to capture crucial sequence distributions and predictive models for effective virtual screening, generative AI (GenAI)-empowered approaches are rapidly emerging as promising methods for de novo promoter design. Both we and other researchers have successfully demonstrated the power of generative models in expanding the scope of accessible sequence space and designing novel promoters with desired functions in *E. coli*, Yeast, and mammalian cells (Kotopka and Smolke 2020, Van Brempt *et al.* 2020, Wang *et al.* 2020, Vaishnav *et al.* 2022, Zrimec *et al.* 2022, Zhang *et al.* 2023).

**Figure 1.**
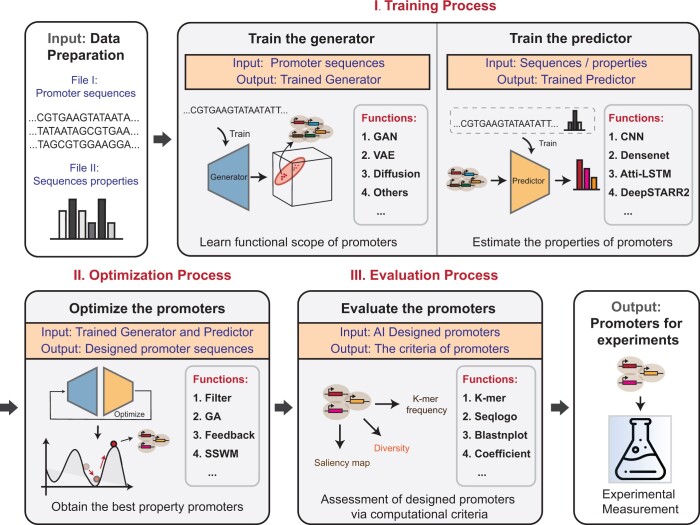
Workflow of GPro toolkit. The scheme illustrates the essential processes of the GPro standardized pipeline. Promoter sequences and activities are given as input to train the generator and the predictor in the training process. Then, combining the trained generator and predictor, the optimizer creates promoters with desired activities. Next, the evaluator assesses the quality of the designed promoters. Finally, promoters designed by GPro can be synthesized for biological experiments.

However, current design methods are highly task-specific and implemented by different programming frameworks. Even researchers with AI expertise have to invest considerable time to adapt these algorithms for new applications, not to mention broader users who may lack advanced programming skills. To address this challenge, we introduce GPro, a user-friendly Python toolkit for synthetic promoter design. This toolkit supports execution with minimal code and can be easily installed on common operating systems, e.g. Windows, Linux, and macOS. We have established a standardized pipeline consisting of training, optimization, and evaluation processes (Fig. 1), which facilitates a user-friendly interface for broader users. Additionally, GPro integrates cutting-edge GenAI-empowered algorithms, providing a suite of selectable algorithms at each process, thereby affording advanced users the flexibility to customize and modularize their promoter design pipelines.

## 2 Implementations

GPro is developed in Python 3.9 and built on the PyTorch framework, enabling standardized input/output for various GenAI-empowered design strategies. Specifically, GPro requires two text files as input: promoter sequences and their corresponding transcriptional activities, which can be quantified by techniques such as Massively Parallel Reporter Assays or RNA sequencing. Input data can be selected from users’ unique scenarios or one of the default datasets we provided. More detailed descriptions of requirements can be found on the online wiki (https://github.com/WangLabTHU/GPro/wiki/).

GPro creates synthetic promoters in a FASTA file and produces a CSV file that lists the GenAI-designed promoters and their predicted activities. Computational criteria are employed to evaluate the quality of the generated promoters. Additionally, the network weights and training reports of the deep neural networks are stored in the relevant generator and predictor folders.

### 2.1 The standardized pipeline for promoter design

We formulated the promoter design pipeline into three parts, as mentioned below (Fig. 1):

#### 2.1.1 Training process

**Training the generator:** The generator’s function is to accurately estimate promoter distributions in the training data, thereby effectively narrowing down the range of sequence possibilities (Arjovsky *et al.* 2017). The GPro generator primarily implements novel sequence generation through models such as Wasserstein Generative Adversarial Network (WGAN), Variational Auto-Encoder (VAE), and Multinomial Diffusion Model (Diffusion). Users have the flexibilities to provide biological data or carefully designed simulated data as the training dataset. Subsequently, users can select a generative model and initiate the training process. In this way, a generator will be developed, capable of producing new sequences that follow the latent distributions of the training data. Users could seek GPro wiki for guidance or directly implement pre-trained models for validation.

**Training the predictor:** The predictor’s function is to estimate the activities of novel promoters for virtual screening. GPro has encapsulated a variety of currently popular promoter strength prediction models from recent publications, including DenseNet, DenseLSTM, CNN_K15, CNN_Wangye, AttnBiLSTM, and DeepSTARR2, supporting the applications of both prokaryote and eukaryotic sequences. Users can provide paired datasets, which consist of sequences and corresponding activity levels to train the predictor. After training, the predictive model will be used to predict the activity levels of sequences generated by the generator. GPro package has also provided guidance on GPro wiki and pre-trained models.

#### 2.1.2 Optimization process

The optimizer’s function is to perform sequence optimization based on the predictor and/or generator. Indeed, the optimizer is conducting a constrained searching process for sequences that enable predictor maximization/minimization. The GPro optimizer covers current mainstream algorithms including virtual screening (filter), genetic algorithm, simulated annealing, gradient descending, feedback, genetic drift, and strong-selection weak-mutation. Users can use pretrained models acquired in the training process, and initiate the optimizer to design promoters towards the desired activities.

It should be noted that the optimization process is not mandatory; users can generate candidate sequences directly through the generator or filter sequences through the predictor. Users have the freedom to combine modules from the generator, predictor, and optimizer to construct their own sequence design pipeline with a high degree of flexibility. We have also performed comparison and guidance of models to help the users to select the most suitable modules for element design.

#### 2.1.3 Evaluation process

The evaluator’s function is responsible for primarily checking the quality of designed promoters, ensuring the Gen-AI designed sequences are well-prepared for biological validations. The GPro evaluator has encapsulated sequence quality criteria (k-mer frequency, blast-n plot, sequence logo, motif discovery) and interpretation methods (saliency map, mutagenesis).

By implementing the above components, GPro offers users a powerful and robust toolkit for promoter design. The effectiveness of GPro toolkit has been validated on several state-of-the-art promoter design tasks (See demo webpage on wiki).

### 2.2 Reference demos

GPro has provided demos for reproducing several cutting-edge promoter design pipelines. First, we reproduced the yeast constitutive promoter design task (Vaishnav *et al.* 2022), utilizing a model with attention mechanisms and bidirectional LSTM to design 110 bp promoter sequences. Second, we presented our previously developed model for the design of constitutive promoters in *E. coli* (Wang *et al.* 2020), which employs a WGAN-GP model as the generator and a convolutional neural network as the predictor to design 50 bp synthetic promoters. In addition, we have replicated two promoter design tasks using the gradient descent algorithm (Zrimec *et al.* 2022) or feedback-GAN (Gupta and Zou 2019). We demonstrated our recent DeepSEED method that integrates prior knowledge using a conditional GAN for inducible promoter design (Zhang *et al.* 2023). Lastly, a demo for tissue-specific enhancer design using a binary classification dataset (de Almeida *et al.* 2023) was provided. It exemplifies the potential for extending the application of GPro to the design of other types of DNA sequences beyond promoters. All demos are readily accessible and can be quickly implemented using the GPro toolkit. Comprehensive details can be found on the “demo” webpage on our online wiki.

In addition, we also offered pre-trained checkpoints for ease of validation. If users need to test the pre-trained models, they can directly load the pre-trained classes through our “generate” and “predict” functions. GPro wiki contains detailed instructions, and all checkpoints and related datasets have been uploaded to Google Drive.

Finally, guidance is provided on GPro wiki for the users who want to integrate customized models or criteria to meet their demands in specific sceneries. We also strongly encourage these users to submit a pull request, and if the method is appropriate, we will incorporate it into the toolkit.

## 3 Discussion

Here, we proposed a user-friendly toolkit, GPro, as an integrated GenAI-empowered toolkit for de novo promoter design. GPro provides implementations of most cutting-edge promoter design methods under the same framework, facilitating the analysis and comparison of diverse approaches and network architectures. Comparing with the general biological sequence designing tools like BioAutoMATED (Valeri *et al.* 2023), which utilize automated machine learning technology to construct models, GPro implements published state-of-the-art promoter design methods proposed by human expertise, aiming to enable users to gain a comprehensive understanding of the design process, and provide detailed guidance for selecting appropriate methods and creating their own pipeline. Our toolkit enables efficient in silico design of promoter sequences, and researchers without AI expertise can easily design promoters for their specific requirements. In addition, we also show that GPro can be extended to design other types of regulatory elements like enhancer. We plan to continuously integrate cutting-edge methods for a wider range of transcriptional regulatory element designs in the future.

## Data Availability

All the code and example data are available at https://github.com/WangLabTHU/GPro/.
